# Use of the Uro Dyna-CT in endourology – the new frontier

**DOI:** 10.1590/S1677-5538.IBJU.2016.0413

**Published:** 2017

**Authors:** Fabio C. Vicentini, Luiz A. A. Botelho, José L. M. Braz, Ernane de S. Almeida, Marcelo Hisano

**Affiliations:** 1 Departamento de Urologia, Centro do Cálculo Renal do Hospital 9 de Julho, São Paulo, Brasil;; 2 Departamento de Anestesia, Hospital 9 de Julho, São Paulo, Brasil;; 3 Departamento de Enfermagem, Centro Cirúrgico do Hospital 9 de Julho, São Paulo, Brasil

**Keywords:** Nephrostomy, Percutaneous, Radiation, Ureteroscopy

## Abstract

We describe the use of the Uro Dyna-CT, an imaging system used in the operating room that produces real-time three-dimensional (3D) imaging and cross-sectional image reconstructions similar to an intraoperative computerized tomography, during a percutaneous nephrolithotomy and a contralateral flexible ureteroscopy in a complete supine position. A 65 year-old female patient had an incomplete calyceal staghorn stone in the right kidney and a 10mm in the left one. The procedure was uneventful and the intraoperative use of the Uro Dyna-CT identified 2 residual stones that were not found by digital fluoroscopy and flexible nephroscopy at the end of surgery, helping us to render the patient stone-free in one procedure, which was confirmed by a postoperative CT scan. Prospective studies will define the real role of the Uro Dyna-CT for endourological procedures, but its use seems to be a very promising tool for improving stone free rates and decreasing auxiliary procedures, especially for complex cases.

## INTRODUCTION

Computerized tomography (CT) is the gold standard for evaluation of residual stones after any endourological procedure, but it is commonly performed after the surgery. The recent development of the Uro Dyna-CT (Siemens Healthcare Solutions, Erlangen, Germany) provides CT-like images in the endourological operating room, in addition to working as a digital fluoroscopy. The use of this new equipment in Urology was described by Ritter et cols. and is still very limited, but might have a major impact if it proves to increase the immediate stone free rates (1, 2). We have reported the first case of the use of the Uro Dyna-CT during a simultaneous percutaneous nephrolithotomy (PCNL) and contra-lateral flexible ureteroscopy (FURS) in a complete supine position.

## SURGICAL TECHNIQUE

We evaluated a 65 year-old female patient, with recurrent urinary tract infections, bilateral lumbar pain for two years and a previous double J stent placement during a renal colic episode for a left proximal ureteral stone. Her body mass index was 36, she had mild hypertension and her ASA Score was 2. CT scan revealed an incomplete staghorn stone in the right kidney of low density (400 Hounsfield Units) (Guy’s Stone Score III) (3) and a 10mm calyceal left kidney stone (380HU). A left ureteral stent was placed previously due to acute renal colic. A proposal of a single stage surgery with the use of the Uro Dyna-CT was done and the patient consented to the procedure. She had a 7-day treatment of 500mg bid ciprofloxacin before surgery and 1g of tranexamic acid at the beginning of the anesthesia.

**Figure 1:**
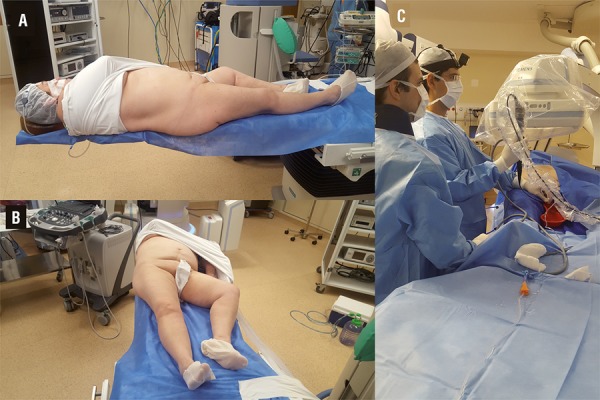
A) Lateral view of the patient in complete supine position; B) Frontal view of the patient in complete supine position; C) Surgical team at the right side of patient performing flexible ureteroscopy and laser lithotripsy.

We performed a right PCNL and left FURS in a complete supine position, as previously described (4) (Figure-1). Briefly, the patient was positioned with the head on the suspended part of the table, under general anesthesia. She was in a supine position, with no boosters under her back. The right side of her back was a few centimeters over the lateral border of the Table, her right leg was straight and her left leg was slightly bent. The surgical table had no option for lithotomy position, but our group is used to performing surgeries in a complete supine position.

**Figure 2:**
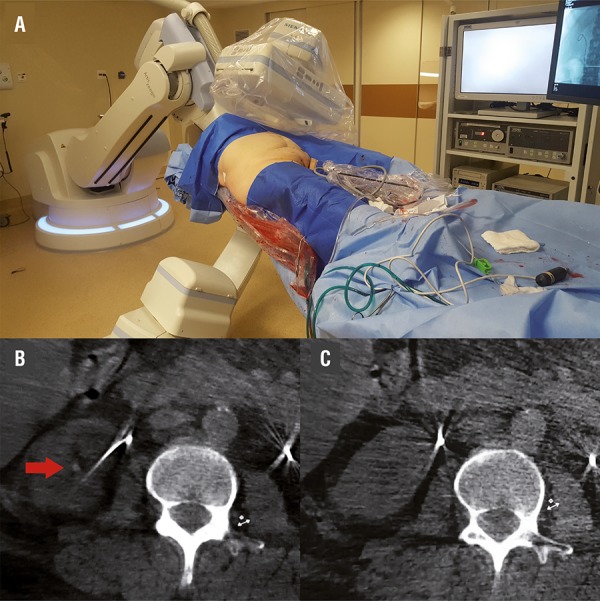
A) Intraoperative image acquisition by Artis Zeego – Uro Dyna-CT; B) Intraoperative CT scan showing one residual stone (arrow); C) intraoperative CT showing a stone free right kidney.

The first step was to make a 3D image for initial evaluation of the stones. Image acquisition was performed using an Artis Zeego (Siemens Healthcare Solutions, Erlangen, Germany) with a standard 6s DCT Body protocol of the Uro Dyna-CT, which gives images of 0.5mm of each slice. We initiated the procedure with a rigid cystoscopy and right side insertion of a ureteral catheter 6Fr. Then, we removed the previous double J stent on the left side and inserted a hydrophilic guidewire inside it. The surgeon was standing all the time on the right side of the patient. A 35cm 12Fr ureteral sheath was inserted and the FURS was performed with a 270nm laser fiber set at 12Hz and 0.5J. Fragments were removed with a tipless nitinol basket and a new double J stent was inserted. At this point, another scan with the Uro Dyna-CT was done, to check the stone status on the left. A 20% diluted contrast agent was used and was still present inside the collector system. Then, with the patient in the same position, a right kidney puncture was done on the previously demarked area on the patient flank, guided by ultrasound. A 30Fr Amplatz sheath, a 26Fr rigid nephroscope and an ultrasonic device were used for treating the stone (Lithoclast Master, EMS, Swiss). After the removal of all fragments, a high resolution fluoroscopy and a flexible nephroscopy were performed and showed stone-free status. A 3D image with the Uro Dyna-CT showed the presence of 4 and 2mm residual stones that were found and removed with the rigid nephroscope, achieving the final stone-free status (Figure-2). Another 3D image was made for final stone-free status checking. A double J stent was retrogradely inserted at the right side and the procedure was tubeless.

**Figure 3:**
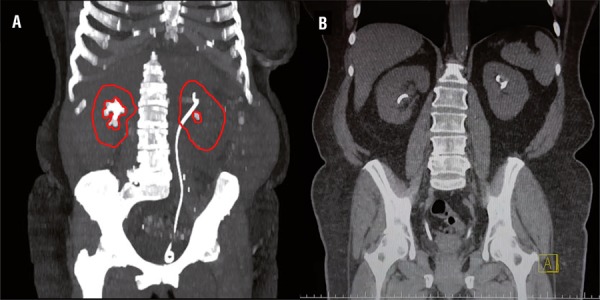
A) preoperative CT scan showing an incomplete staghorn stone in the right kidney of low density and a 10mm calyceal left kidney stone; B) postoperative CT scan showing the stone free status and bilateral ureteral stents.

Total surgical time, from the beginning of the cystoscopy until the removal of the Amplatz sheath was 175 minutes. The hemoglobin drop was 1.6g/dL and the patient was discharged on POD1. A CT scan at the POD2 confirmed no residual fragments on both sides (Figure-3). Stone analysis revealed a pure uric acid composition. The total radiation dose for this initial case was 1912.8mGy (541.9 for preoperative CT, 83.5 for digital fluoroscopy, 745.5 for five 3D images, and 541.9mGy for the final post operative CT).

## COMMENTS

The use of Uro Dyna CT works like an intraoperative CT scan, in addition to producing fluoroscopic images. It gives 0.5mm cross-sectional images and has a high correlation with the real stone size (5). Its use in Urology has been described recently and its indication is not established yet. In our case, it correctly identified two residual stones that were not visualized by a high resolution fluoroscopy and the flexible nephroscopy, helping us to render the patient stone-free in one procedure. This result was very exciting and gives us the expectation that the routine use of the Uro Dyna-CT might improve the outcomes of endourological procedures. Particularly in this case, the stone was radiolucent, and potential residual fragments were very difficult to see. The residual stones images were not clearly seen on the screen as a normal CT and the presence of the guidewire causes some artifacts. The total dose of radiation certainly can be reduced, when we define the moment of making the acquisitions and the best customized protocol. Besides that, if the accuracy of the Uro Dyna-CT for residual stones proves to be similar to a CT scan, we can preclude this postoperative image, reducing the radiation exposure. We opted for not using the Uro Dyna-CT for puncture, because it was an easy puncture and its use could increase the radiation dose unnecessarily.

The procedure was performed in a complete supine position with no difficulties, perhaps because our group has a large experience operating in this position; actually, it was very comfortable for the surgeon and the assistant, because the screens were in the front of the team and no neck twisting was necessary. For those who are not used to this position, it can cause some difficulties in the initial cases. However, if a semi-rigid ureteroscopy is necessary, the table may not be suitable for that. Ritter et cols. described the use of the Uro Dyna-CT for punctures for PCNL in prone position, but the results in terms of stone-free rates are still lacking (2).

Some questions need to be clarified for using this new technology:

1. When should we use it? For all cases or just for complex ones?
2. How many 3-D images do we need?
3. Which is the best imaging protocol?
4. What is the real accuracy for residual stones?
5. Does it really improve the outcomes and reduce reoperations?
6. Are the patients and staff exposed to more or less radiation?
7. Is it cost-effective?

The Uro Dyna-CT in not available worldwide, but can be found in some institutions, mainly for angiographic studies. Prospective studies will verify the real role of the Uro Dyna-CT, but it’s believed that it has a great potential of improving the immediate stone-free rates of complex endourological procedures, reducing the necessity of post operative CT scans and auxiliary procedures.
